# Ethnopharmacological study of native medicinal plants and the impact of pastoralism on their loss in arid to semiarid ecosystems of southeastern Iran

**DOI:** 10.1038/s41598-020-72536-z

**Published:** 2020-09-23

**Authors:** Mohsen Sharafatmandrad, Azam Khosravi Mashizi

**Affiliations:** Department of Natural Science, Faculty of Natural Resources, University of Jiroft, 8th km of Jiroft - Bandar Abbas Road, P.O. Box 7867161167, Jiroft, Iran

**Keywords:** Ecology, Environmental sciences, Environmental social sciences

## Abstract

The purpose of this study was to gather ethnopharmacological information on plants used by the pastorals of southeastern Iran. The relationships between ecological value of the plant species and ethnobotanical indices were investigated. The loss of medicinal plants and its effective factors were also determined under nomadism and sedentary pastoralism. Ethnopharmacological information of plants was collected through interviews with 85 local people including nomads (43%) and sedentary pastorals (57%). Ethnobotanical indices including relative frequency of citation (RFC), relative importance (RI), cultural value (CV), and use value (UV) were estimated. Canopy cover and density of plant species were measured at 60 sampling plots in the exclosure, nomadic rangelands and sedentary pastorals rangelands. The Importance Value Index (IVI) and Relative Loss Index (RL) were estimated for both nomadic and sedentary pastoral rangelands. Pearson correlation coefficient was used to investigate the relationship between ethnobotanical indices and IVI of plant species. The Bayesian networks was used to investigate the relationship between ethnobotanical indices and plant species loss. In total, 156 medicinal plant species of 50 families were identified in the region by locals. Positive correlation was observed between ethnobotanical indices (RFC and RI) and ecological index (IVI). The mean decline of the ecological importance of medicinal species in sedentary pastoral rangelands was approximately three times higher than in nomadic rangelands*.* Bayesian networks showed that cultural value, seed exploitation and aerial parts exploitation had direct relationships with species loss in both nomadic and sedentary pastoral rangelands. *Bunium persicum*, an ecologically and socially important species, had been extensively harvested (more than 60%) in the both nomadic and sedentary pastoral rangelands, making it a priority species in future conservation programs. Medicinal plants had high ecological value and were severely exploited, threatening sustainability of arid and semiarid ecosystems. Local pastorals not only use medicinal plants as herbal remedies but also consider them as a source of income. Popular plants with multiple medicinal uses were more susceptible to loss. Higher medicinal knowledge of pastorals did not help to mitigate medicinal plant loss, requesting new plans to aware them to the circumstances that often leads to species removal from community. Given the importance, abundance and widespread use of medicinal plants, further studies can provide a basis for their conservation and for identifying new therapeutic effects of plants in the region.

## Introduction

Plants provide many uses for humans, the medicinal usage is one of their most important benefits traditionally^1,2^. According to the World Health Organization^3^, more than 80% of people depend on traditional use of medicinal plants for their health in developing countries. Botanical studies have considerably increased in recent years^4,5^. Today there are 20,000 plants used for medicinal purposes in the world^6^ and there are about 8000 plant species in Iran, of which 2300 species are aromatic and medicinal^7^. These medicinal plants are mostly distributed across arid and semiarid rangelands, which are historically used by local pastorals.

The history of pastoralism in Iran (Zagros Mountains) is traced back to 10,000 years ago^8^. During this long time, local pastorals gained a lot of information on medicinal plants and their relation to nature, especially in rangelands. They transferred their experiences to later generations. This knowledge that is accumulated over generations of living in a particular environment, known as native or indigenous knowledge^9^. Indigenous knowledge is based on the perceptions and various insights of local communities on the surrounding environment, land resources and their exploitation. Local communities earned it over the years with numerous trials and errors. Using this knowledge, pastorals have sustainably managed their rangelands for a long time. Therefore, indigenous knowledge of pastorals can have a significant impact on the proper use and management of plant resources in rangelands, and has a valuable role in the conservation and sustainable use of rangelands^10^. The native rangeland management systems also have sophisticated features that reflect the relationship between human adaptation, environmental diversity, land use systems and local decision-making systems^11^. Therefore, indigenous knowledge is important and essential in the management of medicinal plants in rangelands.

On the one hand, the knowledge of pastorals can vary based on environmental conditions, animal husbandry systems and social issues^12^, requesting an investigation with different pastoralism types in the region (nomadism and sedentary pastoralism).

On the other hand, ethnobotanical information is of high cultural importance and of great socio-economic value to researchers, politicians, and the local populations^13^. This knowledge is being eroded due to the decline of custodians of indigenous knowledge and the lack of interest of the younger generation to this knowledge^14^. Therefore, researchers should look for approaches for applying and institutionalizing the indigenous knowledge of different pastorals in the plans and adopted policies.

With the increase in human population and thus their demands, the intensive use of wild plants is increasing^15^, threatening plant species ecological importance in different ecosystems. Inadequate ecosystem management and severe exploitation threaten about 8% of medicinal species^16^. In this regard, this study was done to (1) investigate ethnopharmacology of medicinal plants used by local pastorals in southeastern Iran, (2) assess the relationships between ethnopharmacological information of medicinal plants with their ecological importance, and (3) determine medicinal species loss under two different pastoralism types in the region (nomadism and sedentary pastoralism) and their relationship with ethnobotanical indices.

## Methods

### Study area

Khabr National Park was selected for this study. Khabr National Park is located in, southeast of Iran (28° 25′ to 28° 59′ N and 56° 02′ to 56° 38′ E). The total area is 120,000 ha and the elevation ranges from 1000 to 3845 m a.s.l. Mean annual rainfall is 253.69 mm and mean annual temperature ranges from 13.7 to 25.3 °C. Soils are primarily sandy loam with different depth. About 55% of the area is surrounded by fences and ditches excluding grazing livestock for more than 25 years. The northern plains consist of pure stand of *Artemisia aucheri*, which is grazed mostly under sedentary pastoralism and nomadism. Of the nomad families, 81% use the region rangelands for livestock grazing in spring and summer and migrate to the provinces on the northern shore of the Persian gulf (Hormozgan) in autumn and winter^17^.

### Data collection

#### Ethnopharmacology of medicinal plants

Ethnopharmacological interview was used as the basis for data gathering from 8 nomadic tribes and 10 villages in the study area. Nomadic and sedentary pastorals were surveyed using non-proportional quota sampling^18^. A questionnaire was administered only to people who had knowledge of medicinal plants, through face-to-face interviews. Various data such as ethnopharmacological information including local names, purpose of usage, preparation method, and the plant parts used were obtained through interviews and discussions. Furthermore, respondents age, gender, and educational status were also questioned. The voucher specimens were collected on site and were identified by specialist with the help of available floras^19,20^. The species entries were complemented along with data on taxonomic position (family) and vernacular name.

#### Ethnobotanical indices

Some ethnobotanical indices were measured on the basic of collected ethnobotanical information. We have compared the importance of each species using the following four indices: Use-value (UV), relative frequency of citation (RFC), relative importance index (RI) and cultural value index (CV).

#### Use-value (UV)

A quantitative index used to determine the relative importance of an indigenous plant species, which was calculated as follow:

$$UVs = \Sigma Ui /n$$

where U_i_ is the sum of the total number of use citations by all informants for a given species, n is the total number of informants^21^. A high use value indicates the potential importance of the cited plant species.

#### Relative frequency of citation (RFC)

This index shows the local importance of each species and it is given by the frequency of citation (FC, the number of informants mentioning the use of the species) divided by the total number of informants participating in the survey (N), without considering the use-categories as follow^22^:

$${RFC}_{s}=\frac{{FC}_{s}}{N}=\frac{\sum_{i={i}_{1}}^{{i}_{N}}{UR}_{i}}{N}$$

#### Cultural value index (CV)

This index was measured with multiplying three factors using the following formula:

$${CV}_{s}=\left[\frac{{NU}_{s}}{NC}\right]\times \left[\frac{{FC}_{s}}{N}\right]\times \left[\sum_{u={u}_{1}}^{{u}_{NC}}\sum_{i={i}_{1}}^{{i}_{N}}\frac{{UR}_{ui}}{N}\right]$$

where in the first factor, NUs the number of different uses cited for the species and NC is the total number of use-categories. FCs is the relative frequency of citation of the species in the second factor. Finally, the third factor is the sum of number of participants who mentioned each use of the specie (URui) divided by N^23^.

#### Relative importance index (RI)

This index was calculated using the following formula.

$$\begin{aligned}{RI}_{s}&=\frac{{RFC}_{s(max)}+{RNU}_{s(max)}}{2}\\ {\mathrm{RFC}}_{\mathrm{s}(\mathrm{max})}&=\frac{{FC}_{s}}{\mathrm{max}FC}\\{\mathrm{RNU}}_{\mathrm{s}(\mathrm{max})}& =\frac{{\mathrm{NU}}_{s}}{\mathrm{maxNU}}\\ \mathrm{NUs}& =\sum_{u=ui}^{u=uNC}{UR}_{u}\end{aligned}$$

where RFC_s(max)_ is the relative frequency of citation over the maximum, i.e., and was measured with FCs divided by the maximum value in all the species of the survey. RNU_s_ is the relative number of use-categories over the maximum and it was calculated with the number of uses of the species (NU_s_) divided by the maximum value in survey^24^.

#### Ecological data of medicinal plants

Ecological data were collected in three adjacent sites (inside the park, outside the park including both nomad and sedentary pastoral rangelands). Sites were selected in homogeneous areas with similar topography and ecological properties. The vegetation survey was carried out using 180 nested quadrats, which randomly located in the sites to show the loss of plant species under different pastoralism types. As a constant quadrat size may not be appropriate or all plant species with different sizes, sixty 10 × 10 m quadrats were used to sample trees and shrubs and 1 × 1 m sub-quadrats were used to sample semi-shrubs and herbaceous plants in each site. Canopy cover and individual numbers of plant species were recorded in each plot.

Importance value index (IVI) provides information about the ecological importance of a species in plant communities^25^. IVI was used to determine ecological value of medicinal plant in our study. Since species density, frequency and dominance comprised IVI are sensitive indicators to anthropogenic actives^26,27^. Reaction of species to human exploitation can be revealed with comparing IVI with and without exploiting^28,29^. Relative loss (RL) index was measured to assess ecological situation of medicinal plants under both nomadic and sedentary pastoral system using changes IVI.

Index of RL of species s was calculated using the following formula:

$${RL}_{s}=\frac{{IVI}_{s\_in}-{IVI}_{s\_out}}{{IVI}_{s\_in}}$$

where IVI is the importance value index. Vegetation composition was evaluated by analyzing the frequency, density, dominance and IVI, using the following formula^30,31^.

$$IVI=\frac{\mathrm{Relative frequency }+\mathrm{ Relative density}+\mathrm{ Relative dominance}}{3}$$

Relative frequency = Frequency of a species/frequency of all species * 100, Density = Total no: of individuals of a species/total no. of quadrats studied, Relative density = Number of individuals of a species/number of individuals of all species * 100, and Relative Dominance = Canopy cover of a species/Canopy cover of all the species * 100.

### Data analysis

Bayesian networks **(**BNs) were used to investigate the relationship between ethnobotanical indices and relative loss of species for both pastoralism rangelands. The variations of the probability of species loss was estimated under different scenarios.

BNs are a general way to find important paths in a network that are usually not easily estimated by mathematical equations. The calculations in the BNs are estimated using Bayes' theorem^32^. According to Bayes' theorem, a prior probability represents information about the initial uncertainty of a parameter. However, the posterior probability is estimated using the observed data and its likelihood function to update the uncertainty distribution of the parameters. Bayes' theorem updates probability of each factor in the network as follow^33^:

$$p\left(X=x|Y=y\right)=\frac{p\left(\pi \mathrm{X}=\mathrm{x},\mathrm{Y}=\mathrm{y}\right)}{p(Y=y)}=\frac{p\left(X=x\right)p\left(Y=y|X=x\right)}{\sum_{{x}^{\prime}}p(X={x}^{\prime})p\left(Y=y|X={x}^{\prime}\right)}$$

Sensitivity analyses in BNs was used to determine factors effective on relative loss of medicinal plants under nomadism and sedentary pastoralism^34^ and then, scenarios were defined based on the most effective factors to predict changes in relative loss with altering effective factors.

Pearson correlation coefficient was used to investigate the relationship between ethnobotanical indices and IVI. A t-test was applied to compare relative loss of plant species under nomadism and sedentary pastoralism.

### Ethics approval and consent to participate

All experimental protocols were approved by Review Board of Faculty of Natural Resources, University of Jiroft, Iran. All methods were carried out in accordance with relevant guidelines and regulations. Informed consent was obtained from all participants.

## Results

### Respondents’ socio-demographics

All of the 85 participants who randomly selected in the study (55 men and 30 women) were locals. The participants were belonged to two pastoralism types i.e. nomadism (43%) and sedentary pastoralism (57%). The focus was on older generations as the holders of traditional knowledge. Therefore, 98% of the participants were more than 45 years old and 60% were over 60 years old (Table 1).

**Table 1:** Socio-demographics of the respondents.

Characteristics	Class	Frequency	Percentage
Gender	Female	30	35
	Male	55	65
Age (year)	< 45	2	2
	45–55	8	9
	55–65	25	30
	65 <	50	59
Pastoralism types	Nomadism	37	43
	Sedentary pastoralism	48	57
Education	Less than high school	33	39
	High school	25	30
	Bachelor’s degree	24	28
	Higher degrees	3	3

### Medicinal plants

**Figure 1:**
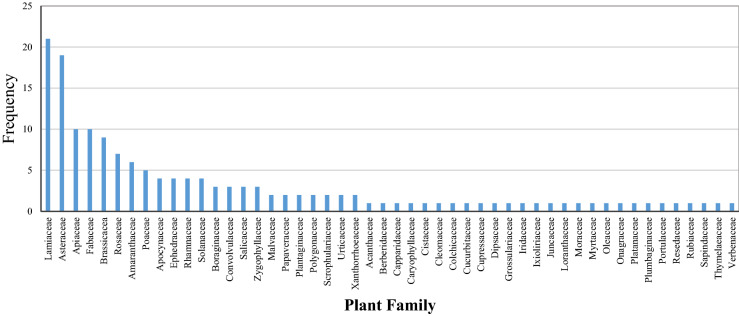
Number of cited plants in each plant family.

In total, 156 medicinal species of 50 families with medicinal uses were identified by pastorals (Table 2). The families Asteraceae, Lamiaceae, Apiaceae, and Fabaceae were the most abundant families in terms of medicinal species in the region (Fig. 1).

**Table 2:** Indigenous medicinal knowledge of plants species in Khabr National Park, Iran.

Scientific name	Family	Local name	Life form	IVI	Plant part used	Medicinal uses	Preparation	Mode of application
*Acantholimon scorpius* (Jaub.&Spach)Boiss	Plumbaginaceae	Khar poshtou	Geophyte	0.001	Flower, Fruit	Sore throat, Dry cough, Removing phlegm throat	Decoction	Oral
*Achillea wilhelmsii* K.Koch	Asteraceae	Bomadaran	Geophyte	0.015	Aerial parts	Diuretic, Tranquilizer, Disinfectant, Anti-flatulence, Analgesic	Decoction, Distillation, Moisturized with water	Oral
*Acinos graveolens* (M.Bieb.) Link	Lamiaceae	Malango	Therophyte	0.001	Aerial parts	Sore throat, Dry cough, Removing Phlegm Throat	Decoction, Moisturized with water	Oral
*Aerva javanica* (Burm.f.) Juss. ex Schult	Amaranthaceae	Pashmouk	Phanerophyte	0.002	Leaves, Flowers	Diuretic, Kidney stone	Decoction	Oral
*Alhagi maurorum* Medik	Fabaceae	Adour	Hemicryptophyte	0.001	Aerial parts	Hemorrhoids; Leaf for rheumatism	Cataplasm, Decoction	Oral, Topical
*Alhagi pseudalhagi* (M. Bieb.) Desv. ex B. Keller & Shap	Fabaceae	Adour	Hemicryptophyte	0.001	Aerial parts	Diuretic, Cathartic, Leaf for rheumatism	Decoction, Liniment	Oral, Topical
*Alyssum dasycarpum* Stephan ex Willd	Brassicaceae	Qodoumeh	Therophyte	0.002	Fruits, Aerial parts	Sore throat, Dry cough, Removing Phlegm Throat	Decoction	Oral
*Amaranthus hybridus* L	Amaranthaceae	Taj-e Khoroos	Therophyte	0.021	Seeds, Flowers, Leaves	Immune System, Brain and nervous system, Headache	Decoction	Oral
*Ammi majus* L	Apiaceae	Golsefid	Therophyte	0.003	Fruits	Nausea, Diuretic	Decoction	Oral
Amygdalus elaeagnifolia Spach	Rosaceae	Archen	Phanerophyte	0.008	Fruits	Swollen Gums, Convulsant, Anemia, menstruation, roots for intestinal worm	Liniment, Decoction,	Oral, Topical
*Artemisia sieberi* Besser	Asteraceae	Dormoun	Chamophyte	0.023	Current year twigs	Anti-angel, abdominal parasites, disinfectant	Decoction, Moisturized with water	Oral
*Asphodelus tenuifolius* Cav	Xanthorrhoeaceae	Peymaouk	Geophyte	0.005	Seeds, Leaves	Diuretic , Swollen Gums, Intestinal worm , rheumatism	Decoction	Oral
*Astragalus crenatus* Schult	Fabaceae	Nakhonak	Therophyte	0.012	Fruits	Colds, Analgesic	Decoction	Oral
*Astragalus gossypinus* Fisch	Fabaceae	Gavan	Chamophyte	0.018	Gum	Heir	Moisturized with water	Oral
*Berberis integerrima* Bunge	Berberidaceae	Zarch	Phanerophyte	0.013	Fruits, Roots	Blood purifier, heat regulation, Edible fruit	Decoction	Oral
*Blepharis edulis* (Forssk.) Pers	Acanthaceae	Khar sonbol	Hemicryptophyte	0.002	Leaves, Seeds	Blood coagulant	Liniment	Topical
*Bunium persicum* (Boiss.)B.Fedtsch	Apiaceae	Zireh-e Siyah	Geophyte	0.022	Fruits	Flatulence, Spasm, Antimicrobial, Menstrual pains, Spice	Decoction	Oral
*Calotropis procera* (Aiton) Dryand	Apocynaceae	Kharak	Phanerophyte	0.001	Leaves, roots, Gum	Leaf for sedative after snake, scorpion and insect bite; roots for gastric discomfort and migraine	Decoction, Dressing	Oral, Topical
*Capparis spinosa* L	Capparidaceae	Kavar	Chamophyte	0.004	Fruits, Roots	Diuretic, cathartic, Antimicrobial, Oickled flower buds	Decoction	Oral
*Capsella bursa-pastoris* (L.) Medik	Brassicacea	Kiseh-e Keshish	Therophyte	0.002	Aerial prts	Blood coagulant	Decoction	Oral
*Chenopodium album* L	Amaranthaceae	Salmak	Therophyte	0.007	Aerial parts	Laxative, Febrifuge	Decoction	Oral
*Chrysopogon aucheri* (Boiss.) Stapf	Poaceae	Rish Zard	Hemicryptophyte	0.005	Roots	antiseptic, repellent and treatment of stomach ache, colds and fever	Decoction	Oral
*Cichorium intybus* L	Asteraceae	Kasni	Hemicryptophyte	0.008	Roots	Diuretic, tranquilizer, febrifuge, diaphoretic, Stomach strengthening, jaundice	Decoction,	Oral
*Cirsium arvense* (L.) Scop	Asteraceae	Kangar saharaei	Hemicryptophyte	0.008	Roots	Gastric discomfort, appetizing	Decoction	Oral
*Citrullus colocynthis* (L.) Schrad	Cucurbitaceae	Hanzal	Therophyte	0.003	Fruits, Aerial parts	Adult squirt, liver cysts, hypertension antipyretic	Decoction, Distillation,	Oral
*Cleome coluteoides* Boiss	Cleomaceae	Alaf-e Mar	Therophyte	0.001	Leaves, Flowers, Fruits	vomiting, Diuretic, cathartic, antiseptic	Decoction	Oral
*Colchicum schimperi* Janka ex Stef	Colchicaceae	Soranjan, Gol-e Hasrat	Geophyte	0.001	Roots	Inflammation and Local pain, gout pains	Decoction, Dressing	Oral, Topical
*Conium maculatum* L	Apiaceae	Showkaran	Geophyte	0.007	Aerial parts	Pertussis, respiratory ailments	Decoction	Oral
*Convolvulus arvensis* L	Convolvulaceae	Pichak	Therophyte	0.014	Aerial parts	Abdominal pains, diarrhea, jaundice, gynecological problem, wound healing,	Decoction, liniment	Oral, Topical
*Convolvulus sericeus* L	Convolvulaceae	Gombeko	Chamophyte	0.015	Current year twigs	Blood purifier, cathartic	Decoction	Oral
*Cotoneaster kotschyi* (C.K.Schneid.) G.Klotz	Rosaceae	Siahchou	Phanerophyte	0.008	Gum	Child squirt, Jaundice	Infusion	Oral
*Cotoneaster persicus* Pojark	Rosaceae	Siahchou	Phanerophyte	0.004	Fruits	Heat regulation	Decoction	Oral
*Cousinia stocksii* C.Winkl	Asteraceae	Siyah Bej	Phanerophyte	0.006	Flowers	milk production in a woman	Decoction	Oral
*Crambe orientalis* L	Brassicaceae	Sepideh	Hemicryptophyte	0.003	Leaves, Flowers	Cytotoxic, antioxidant, antimicrobial and phttotoxic	Decoction	Oral
*Cressa cretica* L	Convolvulaceae	Alaf mourcheh	Chamophyte	0.001	Aerial parts	Antifungal, antibacterial	Liniment	Topical
*Cyanus depressus* (M.Bieb.) Soják	Asteraceae	Gol-e Gandom	Therophyte	0.012	Flowers	cough, Digestive	Decoction	Oral
*Cymbopogon olivieri* (Boiss.) Bor	Poaceae	Nagard	Hemicryptophyte	0.011	Roots	treatment of leprosy, bronchitis and heart disease	Decoction	Oral
*Daphne oleoides* Schreb	Thymelaeaceae	Toorbid	Phanerophyte	0.004	Fruits	cathartic	Drying	Oral
*Descurainia sophia* (L.) Webb ex Prantl	Brassicaceae	Khakshi	Therophyte	0.009	Seeds	Anti diarrhea, Heat regulation	Decoction	Oral
*Dianthus orientalis* Adams	Caryophyllaceae	Gharanphel	Hemicryptophyte	0.008	Flowers	Toothache, nerve tonic, hiccups	Liniment, decoction	Oral, Topical
*Diplotaxis harra* (Forssk.) Boiss	Brassicaceae	Gol Zard	Therophyte	0.007	Aerial parts	anti-inflammatory, anti-bacterial, anti-fungal and anti-tumor	Decoction	Oral
*Dorema ammuniacum* D.Don	Apiaceae	Vosha, Oshtork	Geophyte	0.003	Gum	Infectious wounds, Infection, abscess,	Cataplasm	Topical
*Dorema aucheri* Boiss	Apiaceae	Bile-har, Vosha	Geophyte	0.004	Gum, Leaves	Infectious wounds, Infection, Stomachache	Cataplasm, Liniment	Oral, Topical
*Dracocephalum polychaetum* Bornm	Lamiaceae	Badranj boye	Chamophyte	0.003	Aerial parts	Rheumatism	Decoction, Dressing	Oral, Topical
*Ducrosia anethifolia* (DC.) Boiss	Apiaceae	Meshgak	Hemicryptophyte	0.011	Aerial parts	Stomachache, backache, childbirth pain	Decoction	Oral
*Ebenus stellata* Boiss	Fabaceae	Jou sikhak	Chamophyte	0.011	Flowers	anti-fungal	Decoction	Oral
*Echinops ritrodes* Bunge	Asteraceae	Shekar tigal	Hemicryptophyte	0.004	Fruits	Treatment of digestive disorders, Dry cough	Decoction	Oral
*Ephedra major* Host	Ephedraceae	Ormak	Phanerophyte	0.001	Aerial parts	Analgesic, cold	Decoction	Oral
*Ephedra major* subsp. procera (C.A.Mey.) Bornm	Ephedraceae	Ormak, Rish-e Boz	Phanerophyte	0.001	Aerial parts	Treatment of respiratory diseases	Decoction	Oral
*Epilobium angustifolium* L	Onagraceae	Poneh-e Gavi	Hemicryptophyte	0.011	Aerial parts	Elimination of oral mucositis	Cataplasm	Topical
*Eremurus persicus* (Jaub. & Spach) Boiss	Xanthorrhoeaceae	Serisho	Geophyte	0.011	Flower, Fruits, Roots	Swollen Gums, Swollen eyes, Malaria , blood pressure and blood fat, Edible leaves	Cataplasm , Moisturized with water	Oral, Topical
*Eryngium billardieri* Delile	Apiaceae	Jaz	Hemicryptophyte	0.012	Aerial parts	Removing Phlegm Throat, Bronchodilator, pertussis, spasmodic, Flatulence	Decoction	Oral
*Euphorbia helioscopia* L	Euphorbiaceae	Shirbeng	Geophyte	0.003	Fruits, Roots	Abdominal pains, diarrhea, root for parasite repellent, rheumatism	Decoction	Oral
*Fagonia bruguieri* DC	Zygophyllaceae	Esfand	Therophyte	0.002	Aerial parts	Appetizing, vermicide, carminative	Decoction, infusion	Oral
*Ferula assa-foetida* L	Apiaceae	Anghozeh	Hemicryptophyte	0.003	Gum	Removing Phlegm Throat, Disposal of intestinal parasites	Direct use	Oral
*Ferula oopoda* (Boiss. & Buhse) Boiss	Apiaceae	Koma	Hemicryptophyte	0.002	Gum	Tooth infection, toothache	Cataplasm	Topical
*Fibigia suffruticosa* (Vent.) Sweet	Brassicaceae	Sekei	Therophyte	0.005	Seeds	Headache, sinus infection	Decoction, Powder	Oral
*Ficus carica* L	Moraceae	Hanzir	Phanerophyte	0.004	Leaves, Roots, Fruits, Gum	Leaves for skin diseases; Roots for Disposal of intestinal parasites; Fruits as cathartic, skin burns, Analgesic, Edible fruits	Cataplasm, Powder, Drying	Oral, Topical
*Fortuynia garcinii* (Burm.f.) Shuttlew	Brassicaceae	Makhleseh	Phanerophyte	0.013	Current year twigs	Migraine, sedative, menstruation additive, spasm	Decoction, infusion	Oral
*Fumaria parviflora* Lam	Papaveraceae	Shahtareh	Therophyte	0.011	Current year twig	Treatment for eczema and Cutaneous itching, Diuretic, diaphoretic	Decoction, Dressing	Oral, Topical
*Glycyrrhiza glabra* L	Fabaceae	Motki	Geophyte	0.003	Roots	Removing Phlegm Throat, digestive Disease, increasing blood pressure	Decoction	Oral
*Helianthemum lippii* (L.) Dum.Cours	Cistaceae	Gol Aftabi	Chamophyte	0.001	Current year twigs	analgesic and anti-inflammatory	Decoction	Oral
*Hertia intermedia* (Boiss.) Kuntze	Asteraceae	Kar Qich	Chamophyte	0.003	Leaves	Epilepsy, anti-tumult	Decoction	Oral
*Hyoscyamus reticulatus* L	Solanaceae	Bang Daneh	Hemicryptophyte	0.005	Seeds	Analgesic	Decoction	Oral
*Iris songarica* Schrenk	Iridaceae	Zanbaq	Geophyte	0.005	Roots	Analgesic, Anti-inflammatory	Decoction	Oral
*Ixiolirion tataricum* (Pall.) Schult. & Schult.f	Ixioliriaceae	Gol Baanafsh	Geophyte	0.003	Aerial parts	Stomach Strengthening	Direct use	Oral
*Juncus fontanesii* J.Gay ex Laharpe	Juncaceae	Sazou	Hemicryptophyte	0.013	Roots	Infections	Decoction	Oral
*Juniperus communis* L	Cupressaceae	Overs	Phanerophyte	0.006	Fruits	Stomach Strengthening, Anti-flatulence, appetizing, blood purifier, rheumatism	Powder, Liniment	Oral, Topical
*Krascheninnikovia ceratoides* (L.) Gueldenst	Amaranthaceae	Barg Noghree	Chamophyte	0.004	Roots	skin burns	Cataplasm	Topical
*Lactuca serriola* L	Asteraceae	Kahou khardar	Therophyte	0.013	Leaves	Bone and joint pains, Jaundice, lossing weight,	Liniment	Oral
*Lallemantia royleana* (Benth.) Benth	Lamiaceae	Melango	Therophyte	0.003	Seeds	Cold, cough and Removing Phlegm Throat	Decoction, moisturized with water	Oral
*Lamium album* L	Lamiaceae	Gazaneh	Therophyte	0.013	Leaves	Asthma, cough, antipyretic, osteoporosis, lactiferous	Infusion	Oral
*Launaea acanthodes* (Boiss.) Kuntze	Asteraceae	Charkheh	Hemicryptophyte	0.002	Aerial parts, Gum	Anticonvulsant, sasthma, hemorrhoids, wound healing	Liniment	Topical
*Leonurus cardiaca* L	Lamiaceae	Dom shir	Hemicryptophyte	0.008	Leaves	Cardiac distress	Decoction	Oral
*Lepidium draba* L	Brassicaceae	Mokou	Therophyte	0.003	Leaves, Seeds	Diuretic, Edible leaves	Decoction	Oral
*Levisticum officinale* W.D.J. Koch	Apiaceae	Karfs-e Koohi	Geophyte	0.007	Leaves, Roots	Stomachache, Diuretic, tranquilizer, Bad breath eliminator	Powder, Liniment	Oral, Topical
*Loranthus grewingkii* Boiss. & Buhse	Loranthaceae	Doongi	Epiphyte	0.001	Leaves, Fruits	wounds Healing, heart Strengthening, tranquilizer,	Decoction, Cataplasm	Oral, Topical
*Lycium depressum* Stocks	Solanaceae	Zil	Phanerophyte	0.002	Leaves, Fruits	Epilepsy, squirt, Pertussis	Decoction	Oral
*Lycium shawii* Roem. & Schult	Solanaceae	Div Khar	Phanerophyte	0.003	Fruits	blood Strengthening, Cutaneous itching, toothache, Leaf juice increases visual acuity	Moisturized with water	Oral
*Malva microcarpa* Pers	Malvaceae	Khatmi	Therophyte	0.004	Flowers	Throat protuberance, heat regulation, tooth mass	Moisturized with water , Infusion	Oral
*Malva sylvestris* L	Malvaceae	Khatmi, Panirak	Hemicryptophyte	0.022	Flowers	diaphoretic, heat regulation, oral mucositis, Anti cough	Moisturized with water , Infusion	Oral
*Marrubium vulgare* L	Lamiaceae	Farasiun	Chamophyte	0.002	Aerial parts	Diuretic, Anti-flatulence, appetizing, Anti cough, Anti-venom	Powder, Decoction	Oral
*Medicago sativa* L	Fabaceae	Yonjeh	Chamophyte	0.016	Leaves	nyctalopia, Anemia, cathartic, tranquilizer, Edible leaves	Decoction	Oral
*Melilotus officinalis* (L.) Pall	Fabaceae	Yonjeh-e Zard	Therophyte	0.003	Leaves, Flowers	Diuretic, tranquilizer, Anticonvulsants	Decoction, Infusion	Oral
*Mentha longifolia* ( L.)L	Lamiaceae	Poodaneh, pooneh	Geophyte	0.004	Leaves, Flowers	Stomachache, Anti-flatulence, Spice	Powder, Distillation	Oral
*Myrtus communis* L	Myrtaceae	Moord	Phanerophyte	0.004	Leaves, Fruits	Neuralgia, colds, deodorant, Herpes treatment, Seed as abdominal parasites	Decoction, Powder	Oral
*Nasturtium officinale* R.Br	Brassicaceae	Alaf-e Cheshmeh	Hemicryptophyte	0.005	Aerial parts	Diuretic, tranquilizer, Removing Phlegm Throat, Blood purifier, Neuralgia, Digestive	Direct use	Oral
*Nepeta assurgens* Hausskn. & Bornm	Lamiaceae	Poone say	Chamophyte	0.002	Aerial parts	Diuretic, Anticonvulsants, Anti cough, Disinfectants	Decoction	Oral
*Nepeta bornmuelleri* Hausskn. ex Bornm	Lamiaceae	Badranj boye	Chamophyte	0.005	Aerial parts	tranquilizer, cathartic, rheumatism	Decoction	Oral
*Nepeta glomerulosa* Boiss	Lamiaceae	Chagmal	Chamophyte	0.005	Current year twigs	arthritis	Liniment	Topical
*Nerium oleander* L	Apocynaceae	Gish	Phanerophyte	0.003	Roots	arthritis, Stomachache	Decoction, Liniment	Oral, Topical
*Nonea caspica* (Willd.) G.Don	Boraginaceae	Sezkouei	Therophyte	0.004	Leaves	Cardiac distress, nerve tonic, sedative	Decoction	Oral
*Nonnea persica* Boiss	Boraginaceae	Chezkouei	Therophyte	0.006	Leaves	Sedative	Decoction	Oral
*Ochradenus ochradeni* (Boiss.) Abdallak	Resedaceae	Sham	Chamophyte	0.008	Leaves	wound healing, skin parasites	Liniment	Topical
*Olea ferruginea* Wall. ex Aitch	Oleaceae	Zeytoun-e Koohi	Phanerophyte	0.003	Leaves	Anti allergic, asthma treatment, diaphoretic, Removing Phlegm Throat	Decoction	Oral
*Onopordum leptolepis* DC	Asteraceae	Kangar	Hemicryptophyte	0.002	Aerial parts	Urinary stone, abdominal pains, diarrhea	Decoction	Oral
*Onosma stenosiphon* Boiss	Boraginaceae	Hoochereh	Therophyte	0.008	Roots	arthritis, headache	Decoction, Dressing	Oral, Topical
*Origanum vulgare* L	Lamiaceae	Mirzangou	Chamophyte	0.019	Aerial parts	Diuretic, Anti-flatulence, disinfectant , Analgesic, appetizing, Spice	Powder	Oral
*Papaver dubium* L	Papaveraeae	Khashkhash	Therophyte	0.007	Flowers , Fruits	Analgesic, anti-inflammatory, anti abscess	Drying, Liniment	Oral, Topical
*Parietaria judaica* L	Urticaceae	Goush Mooshi	Chamophyte	0.008	Leaves	Diuretic, Heir, Blood purifier, cathartic, Removing Phlegm Throat	Decoction	Oral
*Peganum harmala* L	Zygophyllaceae	Esfand, Dashti	Therophyte	0.002	Seeds	disinfectant, tranquilizer, abdominal parasites	Drying	Oral
*Pennisetum divisum* (Forssk. ex J.F.Gmel.) Henrard	Poaceae	Berschenk	Hemicryptophyte	0.008	Roots	heart Strengthening, appetizing	Decoction	Oral
*Periploca aphylla* Dcne	Apocynaceae	Gerisheh	Phanerophyte	0.006	Gum	anti-inflammatory, cathartic, wound healing	Liniment	Topical
*Phagnalon rupestre* (L.) DC	Asteraceae	Gol-e Aftabi	Chamophyte	0.004	Aerial parts	Toothache	Liniment	Topical
*Pistacia athlantica* Desf	Anacardiaceae	Baneh	Phanerophyte	0.001	Leaves, Fruits	Diuretic, Menstruation regulation , anti diarrhea, Thirst Quenching, Edible fruits	Direct use, Liniment, Oil	Oral, Topical
*Pistacia khinjuk* Stocks	Anacardiaceae	Kasour	Phanerophyte	0.014	Fruits	Hemorrhoids, stomachache, Anti cough, Jaundice, Backache, Edible fruits	Direct use, Decoction, liniment	Oral, Topical
*Plantago lanceolata* L	Plantaginaceae	Barhang	Hemicryptophyte	0.009	Leaves, Roots, Seeds	Blood diluent, Diuretic, diaphoretic, colds, wound healing	Decoction, Liniment	Oral, Topical
*Plantago major* L	Plantaginaceae	Barhang	Hemicryptophyte	0.001	Seeds	Removing Phlegm Throat, Anti cough,	Decoction	Oral
*Platanus orientalis* L	Platanaceae	Chenar	Phanerophyte	0.001	Leaves	Removing skin patches, hoarseness , sedative after snake bite	Decoction, Dressing, Liniment	Oral, Topical
*Plocama aucheri* (Guill.) M.Backlund & Thulin	Rubiaceae	Karpous	Phanerophyte	0.001	Leaves, Flowers	Facilitating milk digestion in infants	Decoction	Oral
*Pogostemon crassicaulis* (Benth.) Press	Lamiaceae	Zopha	Therophyte	0.008	Aerial parts	Colds, Anti cough, Removing Phlegm Throat	Decoction	Oral
*Populus nigra* L	Salicaceae	Sepidar	Phanerophyte	0.001	Leaves, Current year twigs	Diuretic, Disinfectants, Digestive, Hemorrhagic, Rheumatism, Sciatica, Gout	Decoction, Dressing, Liniment	Oral, Topical
*Portulaca oleracea* L	Portulaceae	Khorfeh	Therophyte	0.006	Aerial parts	Antiseptic, Anti Scurvy , Blood purifier, Thirst Quenching, Intestinal parasites, diaphoretic, muscle relaxant	Direct use	Oral
*Prunus eburnea* (Spach) Aitch. & Hemsl	Rosaceae	Qousk, Arjan	Phanerophyte	0.012	Fruits, Roots	Root for burn treatments, Fruits for neurological pains, Liver colic, Migraine, Rheumatic pains	Decoction, Liniment	Oral, Topical
*Prunus scoparia* (Spach) C.K.Schneid	Rosaceae	Badam-e Koohi	Phanerophyte	0.011	Fruits, Roots	Eczema treatment, wound healing, Edible fruit	Decoction, Cataplasm	Oral, Topical
*Pulicaria gnaphalodes* (Vent.) Boiss	Asteraceae	Kak Koosh	Chamophyte	0.008	Aerial parts	Anti-bacterial , antifungal	Decoction	Oral
*Pycnocycla nodiflora* Decne. ex Boiss	Asteraceae	Sag Dandan	Chamophyte	0.001	Aerial parts	Stomachache	Decoction	Oral
*Rhamnus pallasii* Fisch. & C.A. Mey	Rhamnaceae	Tangras	Phanerophyte	0.022	Skin, Current year twigs, Fruits	Diuretic, cathartic	Decoction	Oral
*Rhamnus persica* P. Lawson	Rhamnaceae	Tangras	Phanerophyte	0.003	Skin, Current year twigs, Fruits	Stomachache, cathartic	Decoction	Oral
*Rhazya stricta* Decne	Apocynaceae	Gish	Phanerophyte	0.002	Fruits, Gum	Toothache, Eye problems	Liniment	Topical
*Rheum ribes* L	Polygonaceae	Rivas	Geophyte	0.005	Stems, Fruits	Stomach Strengthening, blood purifier, Intestinal parasites,	Decoction, Powder	Oral
*Ribes orientale* Desf	Grossulariaceae	Tot-e Roobah	Phanerophyte	0.004	Fruits	Diuretic, cathartic, blood pressure Adjust, Gastrointestinal infection	Decoction, Powder	Oral
*Ricinus communis* L	Euphorbiaceae	Kenton	Phanerophyte	0.005	Seeds	Abdominal pains, diarrhea, emetic	Decoction, Oil	Oral, Topical
*Rosa beggeriana* Schrenk ex Fisch. & C.A.Mey	Rosaceae	Nastaran	Phanerophyte	0.007	Fruits	Colds	Moisturized with water	Oral
*Rumex vesicarius* L	Polygonaceae	Torshak	Therophyte	0.008	Leafs	Appetizing, remove bur from skin, Edible leaves	Direct use, Decoction, liniment	Oral, Topical
*Rydingia persica* (Burm.f.) Scheen & V.A.Albert	Lamiaceae	Goldar	Phanerophyte	0.004	Flowers, Fruits	Toothache, Antimicrobial	Decoction, Cataplasm	Oral, Topical
*Saccharum ravennae* (L.) L	Poaceae	Kash	Geophyte	0.0004	Roots	Diuretic, tranquilizer,	Decoction	Oral
*Sageretia thea* (Osbeck) M.C. Johnst	Rhamnaceae	Toutlangou	Phanerophyte	0.001	Fruits	Blood purifier	Decoction	Oral
*Salix alba* L	Salicaceae	Bid	Phanerophyte	0.004	Leaves, Current year twigs	Burn healing, Wound healing, diaphoretic, Analgesic, Headache, oral mucositis	Distillation, liniment	Oral, Topical
*Salix carmanica* Bornm	Salicaceae	Bid	Phanerophyte	0.003	Leaves, Current year twigs	Burn healing, Wound healing, diaphoretic, Analgesic, Headache, oral mucositis	Distillation, liniment	Oral, Topical
*Salvia macrosiphon* Boiss	Lamiaceae	Moureshk	Hemicryptophyte	0.006	Seeds, Roots	Menstruation additive, Wound healing	Decoction, liniment	Oral, Topical
*Salvia mirzayanii* Rech.f.&Esfand	Lamiaceae	Maryam Goli	Chamophyte	0.003	Aerial parts	Stomachache,	Decoction	Oral
*Sanguisorba minor* Scop	Rosaceae	Toot-e Roobah	Therophyte	0.013	Leaves, Roots	Blood coagulant, Antihomorrhoids, tranquilizer,	Decoction	Oral
*Scabiosa candollei* DC	Dipsaceae	Toosak, Sar banafsh	Therophyte	0.001	Flowers	anti diarrhea, arthritis	Decoction	Oral
*Scrophularia striata* Boiss	Scrophulariaceae	Mokhalaseh	Chamophyte	0.013	Fruits	Gastrointestinal Disorders	Powder	Oral
*Senecio glaucus* L	Asteraceae	Qasedak	Therophyte	0.011	Roots	wound healing	liniment	Topical
*Setaria italica* (L.) P. Beauv	Poaceae	Garch	Therophyte	0.013	Seeds	Flatulence, prevention of hair loss	Decoction, liniment	Oral, Topical
*Solanum alatum* Moench	Solanaceae	Ropask	Chamophyte	0.002	Fruits	Blood coagulant, diaphoretic, Analgesic	Moisturized with water	Oral
*Sonchus asper* (L.) Hill	Asteraceae	Shirtighak	Therophyte	0.005	Leaves	Skin rash	Liniment	Topical
*Sonchus oleraceus* (L.) L	Asteraceae	Shirtighak	Therophyte	0.004	Leaves	Skin ailments	Liniment	Topical
*Sophora mollis* (Royle) Baker	Fabaceae	Zard Gol	Chamophyte	0.006	Leaves, Roots, Seeds	roots for heat regulation and as a diuretic. Leaves and seeds are used for gastrointestinal disorders, urinary tract infections, eczema , abdominal parasites	Decoction	Oral
*Stachys inflata* Benth	Lamiaceae	Sonboleee	Chamophyte	0.007	Flowers, Fruits	Treatment of infectious diseases, rheumatoid arthritis and other inflammatory diseases	Decoction, Powder	Oral
*Stocksia brahuica* Benth	Sapindaceae	Ketour	Phanerophyte	0.001	Seeds	Bone pain, stomachache	Decoction	Oral
*Tanacetum parthenium* (L.) Sch.Bip	Asteraceae	Babouneh	Therophyte	0.007	Aerial parts	Parasite repellent, migraine, anti-inflammation	Infusion	Oral
*Teucrium polium* L	Lamiaceae	Kalpooreh	Chamophyte	0.009	Aerial parts	Stomachache, Anti-flatulence, diaphoretic,	Moisturized with water, Distillation	Oral, Topical
*Thymus fedtschenkoi* Ronniger	Lamiaceae	Ezgen	Chamophyte	0.007	Aerial parts	Stomachache,Anti-flatulence, colds, antiseptic	Decoction, Distillation	Oral
*Tragopogon crocifolius* L	Asteraceae	Sheng	Geophyte	0.009	Gum, Aerial parts, Roots	Gastrointestinal Disorders, Blood coagulant, Wound healing	Direct use, Liniment	Oral, Topical
*Tribulus terrestris* L	Zygophyllaceae	Kharkhasak	Chamophyte	0.012	Fruits	Diuretic, Blood purifier, Kidney stone	Decoction	Oral
*Trifolium pratense* L	Fabaceae	Shabdar	Hemicryptophyte	0.009	Aerial parts	Blood purifier, asthma, bone and joint pains	Decoction, liniment	Oral, Topical
*Urtica urens* L	Urticaceae	Gazaneh	Chamophyte	0.002	Leaves, Stems	arthritis	Liniment	Topical
*Verbena officinalis* L	Verbenaceae	Shahbasand	Therophyte	0.007	Aerial parts	Fever, Nerve tonic	Liniment	Topical
*Veronica anagallis* L	Scrophulariaceae	Sizab	Therophyte	0.011	Aerial parts	Diuretic, stomach strengthening	Decoction	Oral
*Zataria multiflora* Boiss	Lamiaceae	Avishan	Chamophyte	0.005	Aerial parts	Bachache, Gastrointestinal Disorders, Colds, Spice	Decoction, Distillation	Oral
*Ziziphora clinopodioides* Lam	Lamiaceae	Alaleh	Chamophyte	0.008	Current year twig	tranquilizer, stomach strengthening, Colds, brain and nervous system,	Decoction, Infusion	Oral
*Ziziphora tenuior* L	Lamiaceae	Kaakooti	Therophyte	0.008	Aerial parts	stomachache, Antimicrobial and antiseptic, Spice	Decoction, Powder	Oral
*Ziziphus spina-christi* (L.) Desf	Rhamnaceae	Konar	Phanerophyte	0.007	Leaves, Fruits	Colds, Intestinal infections, Heir, Edible fruits	Decoction, Cataplasm	Oral, Topical

### Plant parts used

The plant parts used by pastorals for treatment include stems, flowers, seeds, fruits, roots, gums, leaves, and whole aerial parts, of which aerial parts (25%), leaves (24%), and fruits (24%,) were the most abundant parts used (Table 3). Medicinal uses of the species were divided into 16 different categories, of which the uses for digestive system (50% species), immune system (23% species), sedative (20% species) had the most frequencies (Table 3).

**Table 3:** The percentage of plant species citation in each use category and the plant parts used.

Use category	Frequency	Percentage	Part used	Frequency	Percentage
Digestive system	81	50	Aerial part	39	25
Nervous system	23	14	Leaf	38	24
Skin-hair	33	20	Gum	7	4
Cold-flu-fever	29	18	Root	26	17
Respiratory system	10	6	Fruit	37	24
Flavor-Appetizing	7	4	Seed	16	10
Eye problems	2	1	Flower	19	12
Sedative	34	21	Stem	12	8
Gynecology	9	4			
Cardiac system	4	2			
Musculoskeletal	16	10			
Disorders	15	9			
Blood-wound	32	20			
Liver problems	2	1			
Immune system	38	24			
Food	18	11			

### Ethnopharmacological indices

**Figure 2:**
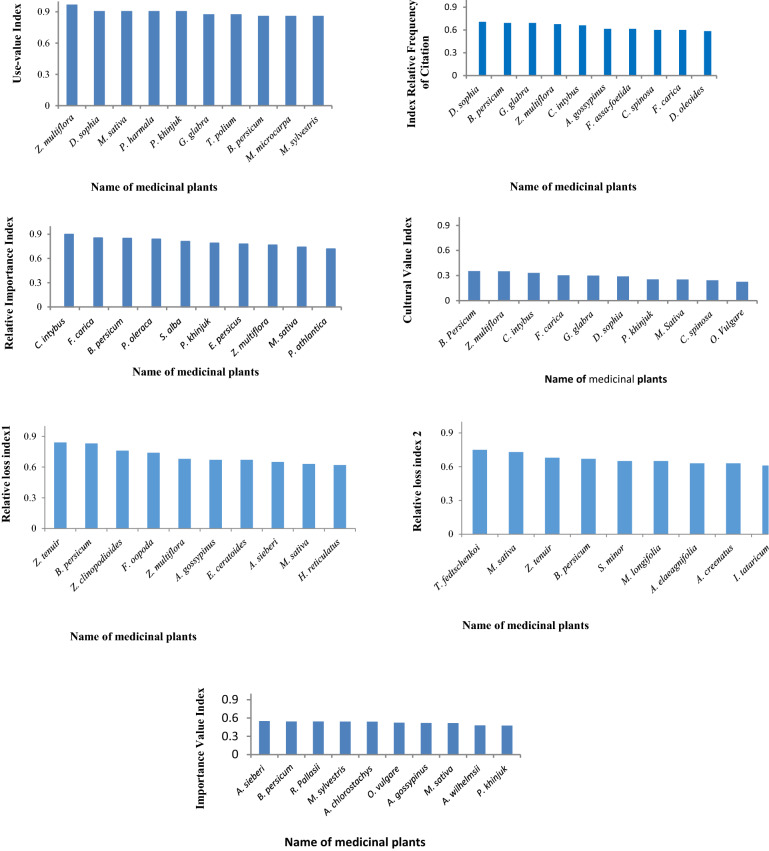
Medicinal plants with the highest ethnopharmacological indices (Use-value, Cultural Value Index, Relative Frequency of Citation, Cultural Importance and Relative Importance and Relative Frequency of Used Plant Parts) and ecological index (relative loss in sedentary pastoralism (RS_1_) and nomadism (RSL_2_) and Importance Value Index).

*Descurainia sophia* (RFC = 0.70), *B. persicum* (RFC = 0.70) and *G. glabra* (RFC = 0.70) had the highest RFC values. UV changed from 0.96 to 0.06 and *Z. multiflora* (UV = 0.96), *D. sophia* (UV = 0.90) and *M. sativa* (UV = 0.90*)* had the highest UVs. The highest IR index belonged to *C. intybus* (IR = 0.89), *F. carica* (IR = 0.85) and *B. persicum* (IR = 0.84). CV changed from 0.35 to 0.004 and *B. persicum* (0.35), *Z. multiflora* (0.35), and *C. intybus* (0.33) had the highest CVs (Fig. 2).

### The relationships between ecological and ethnopharmacological indices

**Figure 3:**
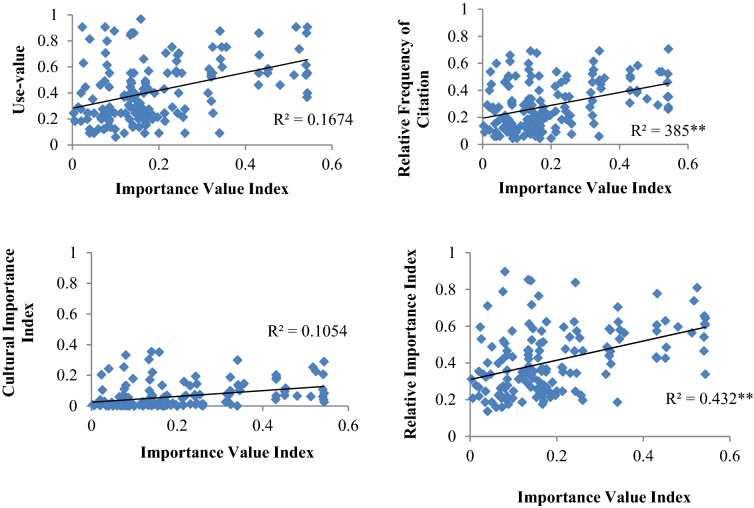
The correlation between Importance Value Index and ethnopharmacological indices (Use-value, Cultural Value Index, Relative Frequency of Citation, Cultural Importance and Relative Importance).

In terms of importance value index, *A. sieberi* (IVI = 0.55), *B. persicum* (IVI = 0.54) and *R. pallasii* (IVI = 0.54) had the highest values in the study area (Fig. 2). Importance value index was significantly correlated with RFC and RI indices (Fig. 3, *p* < 0.01).

**Figure 4:**
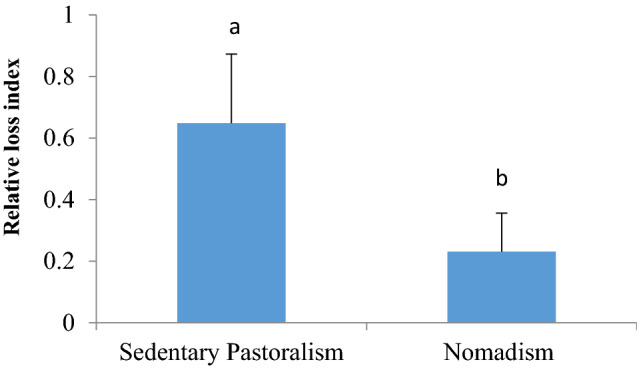
Mean comparison of Relative Loss Index in nomadism and sedentary pastoralism.

The values of relative loss index in the rural area varied between 0.01 and 0.84, and *Z. tenuir* (RL = 0.84), *B. persicum* (RL = 0.83), and *Z. clinopodioides* (RL = 0.76) had the highest values (Fig. 2). However, the values of RL index in the nomadic rangelands varied from 0.03 to 0.75, which *T. fedtschenkoi* (RL = 0.75), *M. sativa* (RL = 0.73) and *Z. tenuir* (RL = 0.68) had the most relative loss index. (Fig. 2). There were significant differences between pastoralism types (sedentary pastoralism and nomadism) in the relative loss of medicinal plant species (Fig. 4). The relative loss indices of medicinal species were 0.648 ± 0.222 and 0.223 ± 0.212 in sedentary pastoral and nomadic rangelands respectively, indicating more species are removed under the sedentary pastoralism.

### Ethnobotanical indices and plant species loss

**Figure 5:**
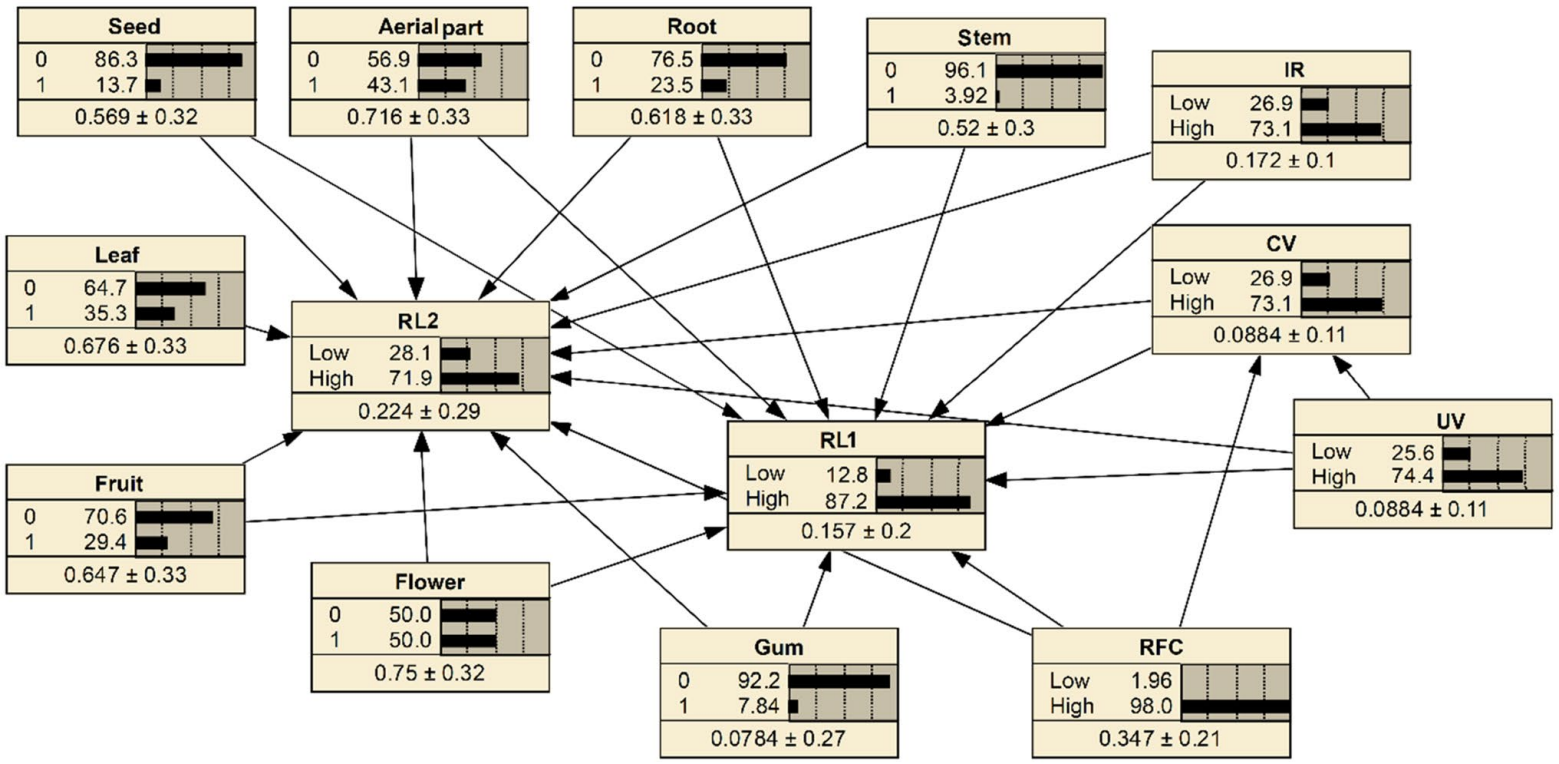
Bayesian networks for assessment of relationships between Relative Loss index of medicinal plants and ethnopharmacological indices in sedentary pastoralism (RL1) and nomadism (RL2) and parts of plant used with prior probabilities.

The Bayesian networks shows the relationship between the relative loss index and ethnopharmacological indices of medicinal plants in sedentary pastoralism (LS_1_) and nomadism (LS_2_) (Fig. 5). There are 15 nodes in the network. The probabilities that relative loss of species being high and low were 87.2% and 12.8% in sedentary pastoralism, respectively. The probabilities that relative loss of species being high and low were 71.9% and 28.1% in nomadism, respectively.

Sensitivity analysis revealed the most important variables affecting the relative loss index (Table 4). The CV, seed, aerial part and RI were the most important variables affecting the relative loss index whose effects on probabilities related to the relative loss were investigated under five different scenarios.

**Table 4:** Effective degree of factors of BNs on Relative Loss Index in sedentary pastoralism (RL_1_) and nomadism (RL_2_).

Relative Loss Index in sedentary pastoralism		Relative Loss Index in nomadism	
Factor	Variance reduction	Factor	Variance reduction
**CV**	**28.31**	**CV**	**19.24**
**Seed**	**25.04**	**Seed**	**17.89**
**RI**	**23.65**	**Aerial part**	**16.13**
**Aerial part**	**21.18**	**RI**	**14.01**
UV	**1.53**	Flower	1.61
RFC	1.13	RFC	1.21
Stem	1.01	Root	0.004
Root	7.05 e−5	Gum	9.24 e−6
Gum	4.17e−5	Leaf	6.62e−6
Leaf	6.83e−6	Stem	3.06e−6
Flower	3.12e−6	UV	6.23e−7

Under scenario 1, by changing the probability of CV index, the effect of increasing this variable on probability of relative loss index was investigated. The results showed that the probability of relative loss was increased to 97.86 and 88.45 for sedentary pastoralism and nomadism respectively, indicating a direct relationship between the relative loss and CV indices. Therefore, harvesting plant organs is one of the main causes of plant loss in both pastoralism types (Table 5). By changing the probability of seed, aerial part, and RI index, the effects of these variables increase on probability of relative loss index was investigated under the scenarios 2 to 4. The probabilities of relative loss index were respectively increased 95.23%, 90.04% and 89.03% in sedentary pastoralism and 83.03%, 78.12% and 75.82% in nomadism under scenarios 2 to 4 (Table5), indicating a direct relationship between the relative loss index and seed, aerial part, and IR index.

**Table 5:** Prior probability and posterior probability of two classes (low and high) for Relative Loss Index in sedentary pastoralism (RL_1_) and nomadism (RL_2_) under 4 scenarios.

	Prior probability	Posterior probability			
		Scenario 1	Scenario 2	Scenario 3	Scenario 4
**RL**_**1**_					
Low	12.8	2.14	4.77	9.96	10.97
High	87.2	97.86	95.23	90.04	89.03
**RL**_**2**_					
Low	28.1	11.55	16.97	21.88	24.18
High	71.9	88.45	83.03	78.12	75.82

## Discussion

The region had a rich medicinal flora, which mostly belonged to Lamiaceae and Asteraceae families. The rich flora can be related to the diverse topo-climatic zones in the region from warm low elevation plains in the south to snow caped mountains and cold high elevation plains in the north, providing habitat for different plant species. Lamiaceae and Asteraceae are two main medicinal families in Iran^35,36^. The dominance of these two families can be attributed to their widespread distribution in the area and their diverse traditional medicinal uses for the pastorals.

Decoction was the most common method of preparation in the region. Decoction is a powerful method for extracting the active ingredients of medicinal plants^37^, making it a pluralistic approach to faster and better treatment between local families^38,39^.

The medicinal plants were mostly used for digestive system between pastorals. Given the high incidence of gastrointestinal diseases among the population, there is more interest in treating it by locals^40^.

Local people mostly used leave of medicinal plants for health care. The leaves are usually easier to harvest than other plant organs and can be usually eaten directly as medicines. Leaves are rich in phytochemicals^41^, resulting in wide medicinal values^42,43^.

Based on ethnopharmacological indices, *Z. multiflora*, *D. sophia*, *C. intybus*, and *B. persicum* were the most important medicinal plant species for the pastorals. These species are completely known as herbs (*Z. multiflora*, *D. sophia*, *C. intybus*, and *B. persicum*) or spices (*Z. multiflora* and *B. persicum*) across the country^44,45^. Therefore, it is obvious they attract locals’ attraction.

### Ecological value of medicinal plants

Our resulted showed that popular medicinal plants had higher dominance and abundance in ecosystem. Past studies also found a positive relationship between ecological value and traditional medicinal use of plant species^46,47^. Common species are more mostly used as medicines than rare species^48^. A number of studies suggested a negative relationship between ecological important of species and its medicinal use^49^.

Medicinal plants *A. sieberi*, *B. persicum* and *R. pallasii* play important role in ecosystem sustainability due to having the highest ecological value. Overexploitation of these species may endanger supplying other medicinal plants, because loss of important ecological species can consecutively destruct the ecosystem balances and influence the dynamics and structure of populations or even drive other species to extinction^50^. Therefore, conservation of such species is more important in ecosystem management than other species.

### Medicinal plants loss and effective factors

Medicinal plants were strongly threated in arid and semiarid ecosystems of the region. About 15% of medicinal plants lost more than 50% of their ecological importance in pastoral rangelands due to overexploitation. Locals are mostly poor people whom their livelihood strongly depends on natural resources in our study area^17^. Selling of medicinal plants has become an alternative and additive income source for pastorals, especially whom with forage deficiency resulted from recent droughts.

We found medicinal plants with higher social value are particularly vulnerable to overexploitation. The harvest of species with high use popularity may encounter sustainability problems^51^. The plant part used and exploitation method can effect on medicinal species loss. Harvesting of plant seed and whole of aerial parts endangered sustainable use of medicinal plants^41,52^. The ecological importance of *B. persicum* as one of the medicinal plants with the highest CV, has declined 80% and 60% in the both pastoral rangelands, due to over-collecting of its seeds. Over-exploitation of aerial pats of *Z*. *tenuir*, *T. fedtschenkoi, M. sativa* and *Z. clinopodioides* has caused depletion of these plants in the both pastoral rangelands. These species were popular medicinal plants with relative importance more than 60% for social system. Reduction of photosynthetic ability linked with the loss of leaf area of plants can restrict plant growth in ecosystems^53^.

Sever loss of medicinal plants has revealed the necessity of serious efforts to create public awareness about value of medicinal plants in aid and semiarid ecosystems of Iran. Gradual loss of traditional knowledge has intensified harvesting of medicinal plants^54,55^. In our study, medicinal plants were conserved more in rangelands under nomadism compared to sedentary pastoralism. Nomadism has less destructive effects on the rangelands due to limited exploitation months compared to sedentary pastoralism in which all year round the rangelands are exploited^56^. Nomads are in fact the real rangelands dwellers who have historically adapted to the rangelands^17^ and have more experiences and information about the medicinal properties on plants^57,58^.

## Conclusions

This study highlights the presence of ethnobotanical knowledge in southeastern Iran and the occurrence of native medicinal species as a key factor in their potential use and locals' attention. Given the abundance and widespread use of medicinal plants, further studies can provide a basis for identifying new therapeutic effects of plants in the region. Popular plants with multiple medicinal uses were more susceptible to loss. Higher medicinal knowledge of pastorals did not help to mitigate medicinal plant loss, requesting new plans to aware them to the circumstances that often leads to species removal from community. Given the abundance and widespread use of medicinal plants, further studies can provide a basis for identifying new therapeutic effects of plants in the region.

## Data Availability

The datasets used and/or analysed during the current study are available from the corresponding author on reasonable request.
